# The impact of public health events on green economy efficiency in the context of environmental regulation

**DOI:** 10.3389/fpubh.2022.996139

**Published:** 2022-09-30

**Authors:** Jingnan Zhou, Yiming Yuan, Zitian Fu, Kaiyang Zhong

**Affiliations:** ^1^China Center for Special Economic Zone Research, Shenzhen University, Shenzhen, China; ^2^School of Economics, Sichuan Agricultural University, Chengdu, China; ^3^School of Economic Information Engineering Southwestern University of Finance and Economics, Chengdu, China

**Keywords:** four-stage SBM-DEA, public health event, green economy efficiency, environmental regulation, China

## Abstract

Public health crises have become one of the greatest threats to sustainable global economic development. It is therefore important to explore the impact of public health events on green economic efficiency. However, few studies have specifically examined the relationship between public health security and green economic efficiency. Based on the relevant data of 30 Chinese provinces from 2011 to 2019, this paper explores the impact of public health on green economic efficiency by establishing a four-stage SBM-DEA model to construct green economic efficiency indicators and using a panel model. A moderating effect model is established to explore the moderating effect of environmental regulation on the impact of public health on green economic efficiency. In addition, this paper examines the heterogeneity of public health impact on green economic efficiency in terms of geographic location, carbon pilot, and transportation level. It is found that, first, public health events have a significant hindering effect on green economic efficiency. Second, environmental regulation has a significant moderating effect on the impact of public health events on green economic efficiency. Third, the impact of public health events on green economic efficiency changes from hindering to facilitating as the intensity of environmental regulation increases. Fourth, the impact of public health events on green economic efficiency is heterogeneous in terms of geographic location, carbon pilot, and transportation level. The above studies have implications for how to balance economic development and environmental protection in case of a public safety event.

## Introduction

Public health events are characterized by sudden outbreaks, great devastation, rapid spread, and a high degree of uncontrollability (1). Whether it is extinct smallpox, the 14 outbreaks of Ebola, or the COVID-19 pandemic, they all have serious personal and economic impacts (2). Public health is a public utility that concerns people's health. At the same time, it is also a prerequisite for achieving sustainable economic development (3, 4). On five occasions, the WHO has emphasized the importance of paying attention to public health emergencies. The Chinese government has also emphasized, in the “Proposal of the Central Committee of the Communist Party of China on the 14th Five-Year Plan for National Economic and Social Development and Visionary Goals for 2035”, the need to “implement the Health China Initiative, improve national health promotion policies, and build a national public health protection network”. In this context, it is urgent to explore the development of a model of economic and environmental benefits that will help everyone (5). When a public health event occurs, investors are pessimistic and green investment drops sharply (6). The rapid decline of green investment will not only discourage the research and development of green technology but also induce the possibility of a financial crisis, which will seriously hinder green economic efficiency (7). At the same time, the occurrence of public health events has caused great harm to human health. The reduction in the quantity and quality of the labor force hinders production activities and reduces the efficiency of the green economy (8, 9). In addition, the occurrence of a public health event directly leads to the stagnation of green industry development, which then generates a chain reaction that hinders the development of upstream and downstream industries and reduces the efficiency of the green economy (10). However, in the context of environmental regulation, the government can appropriately strengthen the intensity of environmental regulation to guide the green transformation of enterprises and promote the innovation of emission-reduction technologies and green technologies (11). In turn, it affects the relationship between public events and green economic efficiency.

The literature studies a large number of factors that affect the efficiency of the green economy, such as technological progress, industrial structure, energy price system, and energy consumption (12–15). However, there is little research on the impact of public safety incidents on green economic efficiency. How do public safety incidents affect green economy efficiency? By what mechanism does a public safety incident affect it? How does the degree of impact differ in different geographical locations, areas with different traffic levels, and carbon pilot and non-carbon pilot areas? These questions have not yet been answered. To explore these issues, this paper first constructs a panel model to study the direct impact of public security incidents on green economic efficiency. Secondly, a moderating effect model is constructed to explore the moderating effect of environmental regulation on the impact of public health on green economic efficiency. Finally, the geographical location, traffic level, and carbon pilot heterogeneity are tested. The results show that the occurrence of public health events will hinder green economic efficiency. Environmental regulation can mediate the relationship between public health and green economic efficiency. The impact of public health events on green economic efficiency is heterogeneous in terms of geographic location, traffic level, and carbon pilot.

The contributions of this paper are as follows: First, previous research lacks the use of the four-stage SBM-DEA model to construct green economic efficiency indicators. In this paper, the investigation of green economic efficiency is more systematic and in-depth, and the definition, measurement, and theoretical research system of green economic efficiency are enriched and improved. Second, it is necessary to discuss the impact of public health events on the efficiency of the green economy from the perspective of environmental regulation. It is here confirmed that the negative impact of public security incidents on green economic efficiency can be mitigated by implementing corresponding environmental regulation policies. Third, it is also necessary to discuss the impact of public security incidents on the efficiency of the green economy from the perspectives of regions, traffic levels, and carbon pilots. This will help the local government to adapt to local conditions and take more efficient measures to promote the efficiency of the green economy. In-depth research on the above issues will help clarify the specific mechanism of the impact of public security incidents on the efficiency of the green economy. Fourth, we perform robustness testing and endogeneity testing by replacing models and explained variables, ensuring that research conclusions are more reliable. Fifth, this paper enriches the theory of sustainable development and improves the targeting of policies to provide a theoretical basis for policymakers to formulate sustainable development strategies and to facilitate sustainable economic development.

The paper is organized as follows: Section Literature review and mechanistic analysis describes the literature and provides details of the mechanistic analysis. Section Variable selection, model construction, and data sources explains data sources, variable definitions, and empirical models. Section Empirical analysis and discussion includes the benchmark empirical results and their analysis. Section Heterogeneity analysis provides details of the heterogeneity test. Section Robustness testing and endogeneity treatment does so for the robustness test. Section Conclusions and insights sets out the conclusions and insights of the research.

## Literature review and mechanistic analysis

The green economy advocates harmony with nature and establishes a positive link between resources, environment, and economic development to create prerequisites and conditions for sustainable economic development (16). Green economy efficiency incorporates the cost of loss of resources and environmental damage into the scope of benefit examination to measure the degree of effectiveness of the green economy (17). At the beginning of 2020, COVID-19 spread around the world, and the GDP of the world's major economies experienced negative growth. In the long term, the “weak foundation” of public health is the crux. In this context, it is important to explore the impact of public health events on the efficiency of the green economy. Public health events can have a significant impact on tourism, human capital, and green investment, and thus significantly affect the efficiency of the green economy.

Public health events have a significant impact on the tourism industry (18). The transportation industry, catering industry, communication industry, and other industries are closely related to tourism. The public health crisis has brought the tourism industry to a standstill. This will also cause the stagnation of the upstream and downstream industries of tourism (19). In addition, the tourism industry has a long recovery time and is greatly affected by the external environment, which further increases the chain reaction of the industry, resulting in a cliff-like decline in the economy and reducing the efficiency of the green economy.

Public health crises can hit the quality and quantity of the workforce by infection, premature death, and restriction of work activities (20). A good public health approach, especially the control of infectious diseases, helps to promote the accumulation of human capital (21). Human capital, in turn, can act on green economic efficiency directly and indirectly. In terms of direct impact, higher levels of human capital can significantly increase the environmental awareness of society as a whole, laying the foundation for achieving green economic development. As the level of education increases, individuals become more environmentally aware. They are more aware of the waste of resources and environmental pollution caused by traditional lifestyles, and thus change their social and economic activities to promote the efficiency of a green economy (22). At the same time, the improvement of human capital level will also have stronger analytical ability and information-seeking ability. On the one hand, green technology research and development activities will be widely supported, and on the other hand, it will guide the establishment of green development-related schemes, thereby promoting green economic efficiency. The occurrence of public health events will lead to a reduction in the level of human capital, which in turn leads to a decline in the efficiency of the green economy. In terms of indirect effects, human capital can influence green economic efficiency through science and technology innovation and improved resource allocation (23, 24). In addition to external influences on the level of science and technology innovation, internal factors of human capital play a more critical role (25). A high level of human capital is more capable of absorbing and digesting frontier technologies and promoting science and technology innovation, and this innovation has significant positive externalities, which in turn drive green economy efficiency in relation to production efficiency and environmental pollution (25). In terms of improving resource allocation, with the rapid development of the social economy, production activities are gradually complicated and integrated. A high level of human capital also means a high level of collaborative ability. Workers with strong collaborative ability can make rational use of knowledge, skills, resources, etc., which promotes the rapid development of sectors with higher technological content. To achieve an effective allocation of resources and improve the efficiency of the green economy, a strong collaborative approach can help transfer labor, capital, and other factors of production from less efficient sectors to more efficient sectors (26). Therefore, when a public health event occurs, the level of human capital decreases, which hinders technological innovation and reduces the efficiency of resource allocation, thereby hindering green economic efficiency.

The occurrence of a public health crisis event often affects investor sentiment, and investors tend to be pessimistic in the short term. If a public health event is never dealt with effectively or even accelerates its spread, it can further exacerbate investors' pessimistic expectations and affect the country's economic confidence (27). This can lead to a huge drop in green investment, which is likely to lead to the outbreak of long-accumulated risks in the financial markets, triggering a crisis in the financial markets and thus affecting the normal functioning of the entire modern industrial system. Green investment can contribute to green economic efficiency through an economic growth effect and environmental protection effect. Regarding the economic growth effect, green investment can drive the development of green industries, which can drive the development of upstream and downstream non-green industries. At the same time, the increase in green investment can expand the scale of social production, enhance the production capacity, increase the effective supply of society, and improve the efficiency of the green economy. Regarding the environmental protection effect, green investment provides more financial support for green industries and promotes the green transformation and upgrading of industries. This green improvement of industries will also stimulate people's awareness of green environmental protection, which will attract more talent to the green industry, bringing an advanced technology level, management tools, etc. to promote green production efficiency. If an investment is made in green R&D activities, such as pollution treatment technology or emission reduction and pollution reduction technology, it will stimulate R&D and innovation for green technology, reducing environmental pollution and enhancing the efficiency of pollutant treatment (28, 29). Furthermore, to attract more green investment, enterprises will increase research and development in green technology innovation, pay more attention to pollution emissions and resource consumption, and build a greener production model (30). In turn, this also improves the efficiency of the green economy. Therefore, a significant decrease in green investment caused by public health events will hinder green economic efficiency.

In the event of a public health crisis event, the development of the tertiary sector stagnates, the level of human capital decreases, green investment decreases significantly, and the development of the green economy is hampered (31). However, at the same time, it also significantly strengthens the government's environmental awareness and the intensity of environmental regulations to reduce the possibility of recurrence of public health crisis events (32). On the one hand, as the intensity of environmental regulation increases, Porter's hypothesis suggests that it will promote technological progress and innovation, making the green economy more efficient (33). On the other hand, enterprises under the dual pressure of public crisis events and mandatory government policies will favor green transformation, choosing to reduce emissions and conserve resources for the sustainable development of enterprises, which in turn promotes green economic efficiency (34). Therefore, in the context of environmental regulation, public health events promote green economic efficiency.

**Figure 1:**
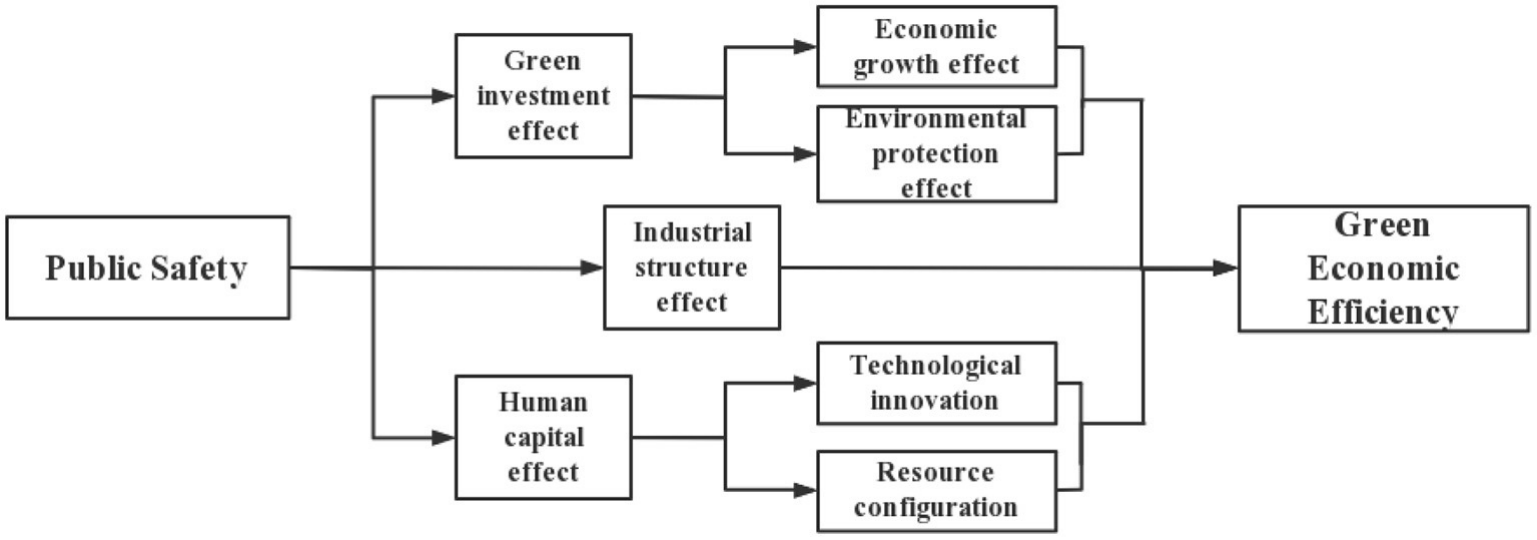
Association and transmission mechanism of public safety and green economic efficiency.

**Figure 2:**
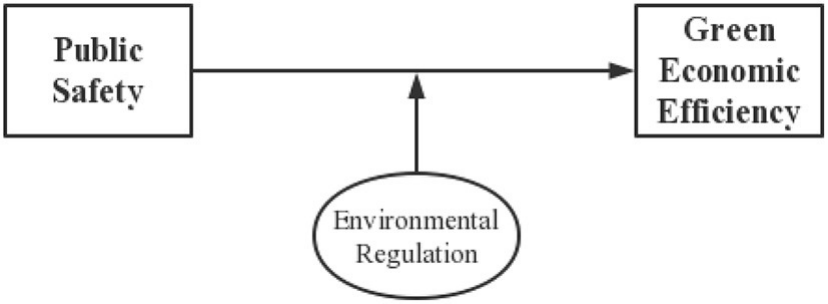
The regulatory role of environmental regulation.

The abovementioned literature provides many theoretical bases for this paper to study the impact of public health on green economic efficiency, but there are some shortcomings. Few studies have incorporated environmental regulation, public health, and green economic efficiency into one framework and examined the transmission mechanism of public health events on green economic efficiency in depth. Against the background of the COVID-19 pandemic and the negative GDP growth of the world's major economies, what measures can be taken to promote the growth of green economic efficiency and achieve sustainable economic development remains to be discussed in depth. To this end, this paper applies a four-stage SBM-DEA to measure green economic efficiency and examines the impact of public health events on green economic efficiency in detail at both theoretical and empirical levels. The transmission mechanism of public health events on green economic efficiency is show in Figures 1, 2.

## Variable selection, model construction, and data sources

### Variable selection

#### Explained variables

In this paper, the energy efficiency index is constructed by a four-stage SBM-DEA.

**Figure 3:**
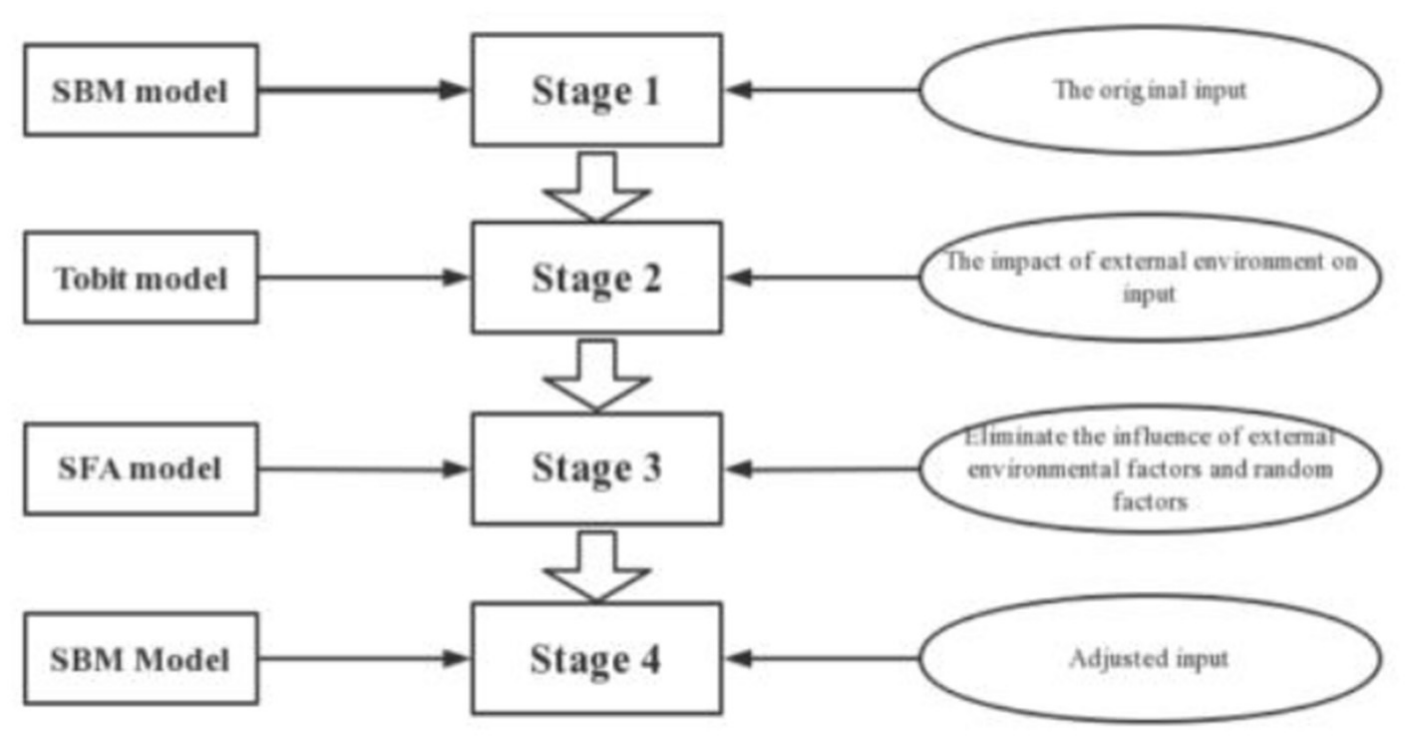
Path diagram of the four-stage DEA model.

Data envelopment analysis is widely used in efficiency evaluation (35). Compared with other efficiency analysis methods, data envelopment analysis can be used to evaluate similar decision units with multiple inputs. Since the traditional DEA model is limited to the same proportional variation of inputs and outputs, the accuracy of the efficiency value of the decision unit will be affected when there is slack in the variables, Tone (36) proposed the SBM-DEA model in 2001, which can solve the problem of slack variables. In a study from 2002, Tone (37) proposed the SE-SBM model to solve the problem of not being able to further evaluate the SBM-DEA model when there are multiple valid decision units. However, the SE-SBM model does not determine whether the efficiency of a decision unit is affected by uncontrollable factors such as external environment and random disturbances, so Fried et al. (38) proposed a three-stage DEA model in 2002. The four-stage DEA constructs a Tobit regression model based on the results of the three-stage DEA model. To measure energy efficiency more accurately, a four-stage SBM-DEA model is constructed in this paper. The path of the four-stage DEA model is shown in Figure 3.

Stage 1: Input slack and initial energy efficiency values are measured for each decision unit. Since the green economic efficiency is expected to be improved by changing the factor inputs, the input-oriented model is chosen in this paper. Assuming that there are n decision-making units (DMUs), each DMU has m inputs and r outputs, and none of the inputs or outputs is less than zero, the model is

(1) $$\begin{array}{l} \min\rho_{0}=1-\frac{1}{m}\overset{m}{\underset{i=1}{\sum }}\frac{\bar{s_{i}}}{x_{i0}} \end{array}$$

(2) $$\begin{array}{l} s.t.x_{i0}=\overset{m}{\underset{j=0}{\sum }}x_{ij}\lambda_{j}+\bar{s};y_{r0}\leq \overset{n}{\underset{j=0}{\sum }}y_{kj}\lambda_{j} \end{array}$$

where ρ is the efficiency metric; m and k are the input and output factor types, respectively; λ represents column vectors; and *x*_0_ and *y*_0_ are the input and output vectors of the decision unit to be evaluated, respectively. *x*_*i*0_, *y*_*i*0_ are the elements of *x*_0_, *y*_0_, $\bar{s}$ is the input slack quantity. The larger the input or output slack, the lower the efficiency value; when the input–output slack is 0, the efficiency value is equal to 1.

Stage 2: Tobit model. After the measurement of efficiency values in the first stage, the amount of slack in the inputs and outputs of each decision unit can be obtained. However, the slack quantity has a value greater than or equal to 0, and there is a truncation in the data when it is equal to 0. Therefore, this paper uses the Tobit truncation model to fit the relationship between input slack quantity and environmental variables in the first stage. Three Tobit regression models are constructed, and the models are defined as follows.

(3) $$\begin{array}{l} s_{ik}=\alpha_{i}+\beta_{i}Z_{ik}+\mu_{i} \end{array}$$

where i is the number of inputs, *i* = 1, 2, ..., *I*; *k* = 1, 2, ....*n*. *s*_*ik*_ represents the total slack of the ith input factor calculated in the first stage, and *Z*_*ik*_ is the vector of external environment variables, and α_*i*_ is the constant term, and β_*i*_ is the vector of coefficients to be estimated, and μ_*i*_ is the random disturbance term.

Stage 3 Decompose the input slack values obtained in one stage using stochastic frontier model (SFA). Construct the regression Equation (2) to decompose the initial input slack values for each decision unit:

(4) $$\begin{array}{l} s_{ik}=f\left(z_{j},\beta_{i}\right)+v_{ij}+\mu_{ij}\;,j=1,2,...j\;.i=1,2,...i \end{array}$$

In Equation (2), *s*_*ik*_ denotes the slack value of the i-th input of the j-th decision unit, *z*_*j*_ is the external environment variable, β_*i*_ is the coefficient of the external environment variable, *v*_*ij*_+μ_*ij*_ is the mixed error term, *v*_*ij*_ denotes the random error term, and μ_*ij*_ denotes the management inefficiency term.

After using Frontier 4.1 to obtain the regression results, the input quantity of the relatively fully effective decision unit was used as the benchmark, and the input quantity of other relatively ineffective decision units was further adjusted by using the regression results to increase the input quantity of the decision unit in a better external environment to reduce the input quantity of the decision unit facing a worse external environment and probability. The specific method is as follows:


(5) $$\begin{array}{l} x_{ij}{\;}^{A}=x_{ij}+[max(f(z_{j},\hat{\beta_{i}})-f(z_{j},\hat{\beta_{i}})]+[maxv_{ij}-v_{ij}],\\ \begin{array}{l} \;j=1,2,...j.i=1,2,...i \end{array} \end{array}$$

where X${\;}_{\text{iJ}}^{\text{A}}$ is the adjusted input, X_ij_ is the pre-adjusted input, and $\max(f(z_{j},\hat{\beta_{i}})-f(z_{j},\hat{\beta_{i}})$ is the adjustment for external environmental factors, and max*v*_*ij*_−*v*_*ij*_ is the adjustment of the random disturbance terms to the same state for all decision units.

Stage 4 Measurement of adjusted SBM efficiency. The adjusted inputs and initial outputs are measured again using the SBM model to derive new efficiency values. Since the interference of external environmental variables is removed in the fourth stage, the efficiency values output in the fourth stage more accurately reflect the actual green economy efficiency level of each decision unit.
  The specific indicators were selected. The green economic efficiency establishment indicators are shown in Table 1.

**Table 1:** Variable settings and descriptive statistics.

**Variables**		**Name**	**Definition**	**Unit**	**Processing**	**Max**	**Min**	**Average**	**Stv**
Input variables	Employment	JOB		10,000 people	Take logarithm	8.875	5.734	7.655	0.782
	Fixed assets	FIX	Amount of investment in fixed assets	Millions of RMB	Take logarithm	15.592	11.874	14.110	0.794
	Energy consumption	ENE	Total energy consumption	Million tons of standard coal	Take logarithm	10.631	7.378	9.428	0.650
Output variables	GDP	GDP		RMB 100 million	Take logarithm	11.587	7.421	9.800	0.853
	Carbon dioxide emissions	CO2		Million tons	Take logarithm	6.843	3.553	5.586	0.725
	Sulfur dioxide emissions	SO2		Million tons	Take logarithm	5.208	−1.661	3.362	1.207
Environment variables	Industry structure	INS	Secondary industry value added/GDP			0.590	0.162	0.440	0.087
	Energy mix	STR	Coal consumption/Primary energy consumption			96.440	0.265	4.611	12.431
	Urbanization rate	CITY	Number of urban population/Total population			0.896	0.350	0.576	0.122
	R&D investment	TEC	Amount of R&D investment	Million yuan	Take logarithm	16.957	10.964	14.280	1.344

##### Input–output indicators

Referring to the literature on green economic efficiency indicators measures (39), we consider capital, labor, and energy as input variables and CO2 emissions as non-desired outputs. Gross domestic product (GDP) is taken as the desired output. In this paper, the number of employees at the end of the year in each province is taken as labor input, fixed asset investment is used as capital input, and energy consumption is used as energy input.

##### External environmental factor indicators

In this paper, industrial structure, energy structure, urbanization rate, and R&D investment are selected as environmental variables. Dong (40) used a spatial econometric model to discuss the relationship and transmission mechanism between technological innovation and green economic efficiency from the perspective of natural resources and urbanization, and concluded that R&D investment will significantly affect green economic efficiency. Song et al. (41) explored the temporal and spatial evolution characteristics of green economic benefits in the Yangtze River Economic Belt by dividing into three watersheds, and found that industrial structure would have a significant impact on green economic efficiency. Bilgen (42) suggested that the energy structure will significantly affect environmental pollution, which in turn affects the efficiency of the green economy. When people migrate between urban and rural areas, it will cause differences in energy consumption, which in turn affects the efficiency of the green economy (43). Based on data availability, this paper uses the ratio of secondary industry added value to GDP, the ratio of coal consumption to primary energy consumption, the ratio of urban population to total population, and R&D investment to measure industrial structure, energy structure, and urbanization rate, all of which are R&D investment indicators.

#### Core explanatory variables

The core explanatory variable in this paper is public health. In this paper, the mortality rate of legal A and B infectious diseases is selected as the main indicator of public health. In the new infectious disease control law, infectious disease are divided into three categories, A, B, and C. There are 39 types of infectious disease, of which infectious diseases in category B are currently not effectively controlled. Since infectious diseases in category A (plague and cholera) are often considered important factors in public health emergencies, the mortality rate of infectious diseases in categories A and B is selected as a measure in this paper. Referring to the analysis of Lumley and Daly (44), it is considered that the mortality rate of statutory infectious A and B diseases as the explanatory variable is relevant and realistic.

#### Control variables

Li et al. (15) believe that the intensity of government funding for science and technology and energy consumption will have a significant impact on the efficiency of the green economy. The government can increase its investment in green technology innovation and improve the energy consumption structure to enhance the green economy. Li et al. (45) believe that energy prices will have a significant impact on the environment. With a rise in energy prices, environmental pollution will be reduced, but a decline in energy prices will distort environmental pollution, which in turn will affect the efficiency of the green economy. Zhang et al. (46) believe that the intensity of foreign trade will have a significant impact on the efficiency of the green economy, and continuously expanding the scale of high-quality foreign trade can promote regional green growth. Therefore, this paper selects government funding intensity, energy consumption, energy prices, and foreign trade intensity as control variables. Based on data availability, this paper uses the ratio of government spending on science and technology to the government's general budget spending to measure the intensity of government funding. The foreign trade intensity is measured by the ratio of total imports and exports to GDP. Energy consumption is measured in terms of energy consumption. Energy prices are measured using the fuel price index in the retail commodity price index.

### Model construction

#### Baseline regression model

Fixed effects models can capture individual heterogeneity in panel data. The fixed effects model uses panel data to expand the sample size, can describe the individuality and commonality between individuals, and improves the estimation accuracy of the model. The model is set as follows:


(6) $$\begin{array}{l} \text{GE}{\text{E}}_{\text{it}}={\text{a}}_{1}+{\text{a}}_{2}\text{PH}{\text{S}}_{\text{i},\text{t}}+{\text{a}}_{3}{\text{Z}}_{\text{i},\text{t}}+{\text{u}}_{\text{i}}+{\text{ε}}_{\text{i},\text{t}} \end{array}$$

where GEE denotes green economic efficiency, PHS denotes public health indicators, Z denotes control variables, u is an individual fixed effect, ε denotes error term, i denotes the province, and t is the year.

#### Moderating effect model

To further test the moderating effect of environmental regulation on the impact of public health on green economy efficiency, a moderated effect estimation model is constructed as follows:


(7) $$\begin{array}{l} \text{GE}{\text{E}}_{\text{it}}={\text{b}}_{1}+{\text{b}}_{2}\text{PH}{\text{S}}_{\text{i},\text{t}}+{\text{b}}_{3}\text{GU}{\text{I}}_{\text{i},\text{t}}+{\text{b}}_{4}{\text{Z}}_{\text{i},\text{t}}+{\text{u}}_{\text{i}}+\varepsilon_{\text{i},\text{t}} \end{array}$$

(8) $$\begin{array}{l} {\text{GEE}}_{\text{it}}={\text{c}}_{1}+{\text{c}}_{2}{\text{PHS}}_{\text{i,t}}+{\text{c}}_{3}{\text{GUI}}_{\text{i,t}}+{\text{c}}_{4}{\text{PHS}}_{\text{i,t}}^{*}{\text{GUI}}_{\text{i,t}}\\ \;+c_{5}Z_{i,t}+u_{i}+\varepsilon_{i,t} \end{array}$$

where GUI denotes environmental regulation, with the interaction term $\left(PHS_{i,t}^{*}GUI_{i,t}\right)$ coefficient to measure the effect of regulation.

### Data source

The data on mortality rates for A and B infectious diseases used in this paper are from the China Health Statistics Yearbook. The data on employment, SO2 emission, secondary industry value added, GDP, and energy consumption were obtained from local statistical yearbooks, the data on total energy consumption were obtained from the China Energy Database, and the data on fixed asset investment, coal consumption, and primary energy consumption were obtained from a wind database. The data on R&D investment is from the China Statistical Yearbook on Science and Technology. Data on urban population, total population, investment in industrial pollution control, government expenditure on science and technology, general budget expenditure, and total imports and export are from the China Statistical Yearbook. The data on carbon emissions are from the China Carbon Accounting Database.

Combined with data availability, the sample interval selected in this paper is 2011-2019, and the panel data used in this paper cover 30 provinces due to the absence of some data from the Tibet Autonomous Region as well as Hong Kong, Macao, and Taiwan. The descriptive statistics of each indicator are shown in Table 2.

**Table 2:** Variable settings and descriptive statistics.

	**Variable**	**Name**	**Definition**	**Processing**	**Max**	**Min**	**Average**	**Stv**
Explained variables	Green economy efficiency	GEE			1.000	0.807	0.913	0.063
Explanatory variables	Public safety	PHS	The mortality rate of A and B infectious diseases		0.082	0.002	0.013	0.015
Adjustment variables	Environmental regulation	GUI	Industrial pollution control completed investment/secondary industry value added	Take logarithm	5.502	0.254	3.106	0.809
Control variables	Government funding intensity	GOV	Government expenditure on science and technology/general budget expenditure		0.066	0.004	0.020	0.014
	Energy consumption	CON	Energy consumption	Take logarithm	11.267	4.055	8.599	1.461
	Foreign trade intensity	OPE	Total imports and exports/GDP		0.240	0.002	0.042	0.047
	Energy prices	PRI	Fuel price index in the retail commodity price index		4.734	4.425	4.619	0.069

## Empirical analysis and discussion

### Energy efficiency evaluation in China based on four-stage SBM-DEA model

#### Phase I measurement results

**Figure 4:**
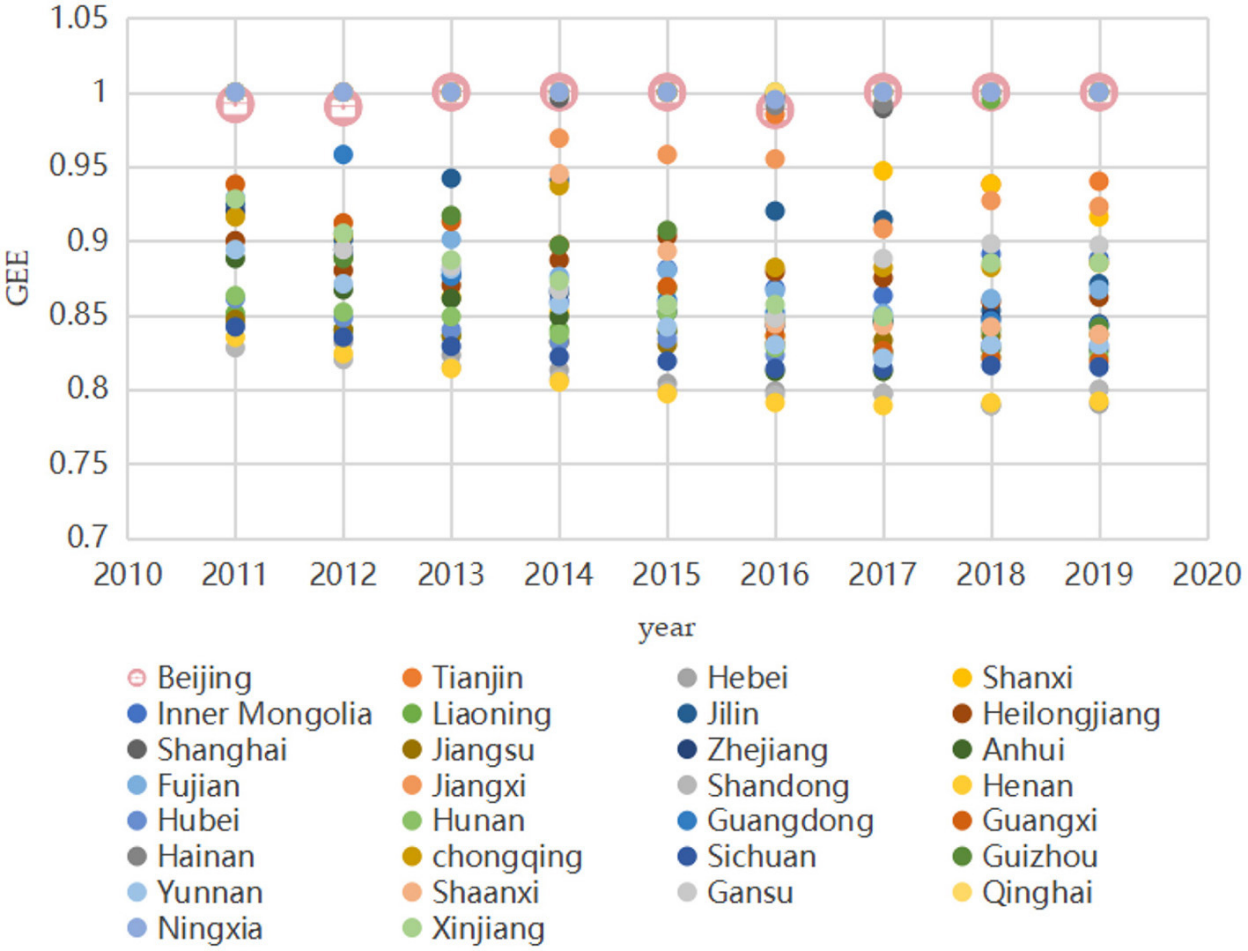
Phase I green economy efficiency.

This paper empirically analyzes the input–output efficiency of the green economic efficiency at the inter-provincial level based on the SBM-DEA model using Stata 16.0 software. The higher value of green economic efficiency represents the superior ability of the province and city to utilize the available resources. The results are shown in Table 3. Figure 4 shows the green economic efficiency of 30 Chinese provinces from 2011 to 2019 in the first stage.

**Table 3:** Green economic efficiency phase I.

	**2011**	**2012**	**2013**	**2014**	**2015**	**2016**	**2017**	**2018**	**2019**	**Average**
Beijing	0.992	0.990	1.000	1.000	1.000	0.988	1.000	1.000	1.000	0.997
Tianjin	1.000	1.000	1.000	1.000	1.000	0.985	1.000	0.938	0.940	0.985
Hebei	0.844	0.832	0.823	0.813	0.804	0.799	0.797	0.789	0.790	0.810
Shanxi	1.000	0.889	0.862	0.852	0.840	0.836	0.947	0.938	0.916	0.898
Inner Mongolia	1.000	0.890	0.880	0.859	0.881	0.868	0.863	0.891	0.888	0.891
Liaoning	0.851	0.840	0.836	0.840	0.868	1.000	1.000	0.995	1.000	0.914
Jilin	0.925	0.901	0.942	0.941	1.000	0.920	0.914	0.853	0.871	0.919
Heilongjiang	0.900	0.880	0.870	0.887	0.903	0.879	0.875	0.859	0.862	0.879
Shanghai	1.000	1.000	1.000	0.996	1.000	0.997	0.989	1.000	1.000	0.998
Jiangsu	0.847	0.840	0.836	0.832	0.830	0.831	0.833	0.836	0.837	0.836
Zhejiang	0.920	0.894	0.877	0.865	0.852	0.843	0.845	0.848	0.844	0.865
Anhui	0.888	0.867	0.861	0.849	0.839	0.812	0.812	0.827	0.826	0.842
Fujian	0.929	0.905	0.901	0.876	0.880	0.866	0.851	0.861	0.867	0.882
Jiangxi	1.000	1.000	1.000	0.969	0.958	0.955	0.908	0.927	0.923	0.960
Shandong	0.828	0.820	0.815	0.807	0.799	0.796	0.797	0.789	0.800	0.806
Henan	0.835	0.824	0.814	0.805	0.797	0.791	0.789	0.791	0.792	0.804
Hubei	0.861	0.848	0.840	0.832	0.834	0.823	0.824	0.830	0.829	0.836
Hunan	0.863	0.852	0.849	0.837	0.852	0.828	0.825	0.822	0.822	0.839
Guangdong	1.000	0.958	0.876	0.867	0.860	0.852	0.847	0.846	0.843	0.883
Guangxi	0.938	0.912	0.913	0.897	0.869	0.836	0.826	0.821	0.819	0.870
Hainan	1.000	1.000	1.000	1.000	1.000	0.991	0.992	1.000	1.000	0.998
Chongqing	0.916	0.904	1.000	0.937	1.000	0.882	0.882	0.882	0.885	0.921
Sichuan	0.842	0.835	0.829	0.822	0.819	0.814	0.814	0.816	0.815	0.823
Guizhou	1.000	0.888	0.917	0.897	0.907	0.847	0.846	0.840	0.842	0.887
Yunnan	0.894	0.871	0.881	0.857	0.842	0.830	0.821	0.830	0.830	0.851
Shaanxi	1.000	1.000	1.000	0.945	0.893	0.844	0.843	0.842	0.837	0.912
Gansu	1.000	0.894	0.883	0.867	0.857	0.848	0.888	0.898	0.897	0.892
Qinghai	1.000	1.000	1.000	1.000	1.000	1.000	1.000	1.000	1.000	1.000
Ningxia	1.000	1.000	1.000	1.000	1.000	0.995	1.000	1.000	1.000	0.999
Xinjiang	0.928	0.905	0.887	0.873	0.857	0.857	0.849	0.885	0.885	0.881

From the results of the first stage of efficiency analysis, the following can be ascertained. Without considering environmental variables, the green economic efficiency of all 30 provinces in China from 2011 to 2019 reached 0.8 or more. The regional differences in green economic efficiency among provinces are large, among which Qinghai has a green economic efficiency of 1 in all 9 years. Henan has the lowest average green economic efficiency. The first stage is only an efficiency evaluation under the traditional SBM-DEA model, and although the SBM model can distinguish between effective decision units, it contains interference from environmental and random factors. Therefore, the green economic efficiency measured by the SBM model alone is undoubtedly unrealistic, and the SFA model needs to be applied to remove the influence of external environmental factors.

#### Second-stage tobit regression results

The slack variables of each input variable obtained in the first stage were used as dependent variables and the four environmental variables were used as independent variables, and the Tobit model was constructed using Stata 16.0 software for analysis, and the results are shown in Table 4. It can be seen that the redundancy values of the three environmental variables on labor, capital, and energy inputs are significant at the 1% level, and industrial structure and R&D inputs pass the 1% test with positive correlation coefficients, indicating that upgrading industrial structure and increasing R&D inputs can make full use of input factors. The energy structure passed the 1% test and the correlation coefficient was negative, indicating that improving the energy structure can make full use of the input factors, that is, environmental factors have a significant impact on green economy efficiency. The first-stage efficiency value cannot fully reflect the current situation of green economy efficiency in each province, and it is necessary to separate the influence of environmental factors to improve the accuracy of the evaluation.

**Table 4:** Tobit regression results.

**Variables**	**Labor input redundancy**		**Capital investment redundancy**		**Energy input redundancy**	
	**Coefficient**	**t-value**	**Coefficient**	***t*-value**	**Coefficient**	***t*-value**
INS	0.838^***^	2.660	1.895^***^	7.360	2.050^***^	6.850
STR	−0.014^***^	−9.720	−0.015^***^	−11.760	−0.008^***^	−6.170
CITY	−0.829	−0.500	−0.780	−0.590	−0.545	−0.370
TEC	0.449^***^	22.580	0.456^***^	24.650	0.401^***^	23.240
C	1.459^*^	1.680	7.588^***^	11.340	3.462^***^	4.370
*F*-value	30.180		58.570		23.510	
Log-likelihood	−124.311		−78.809		−95.589	
r2	0.607		0.754		0.641	

* and

*** indicate significance at the 10, 5, and 1% levels, respectively.

#### Phase III SFA regression results

Using the SFA model with the slack variables of labor, energy, and capital inputs obtained in the first stage as explanatory variables and the four environmental variables as explanatory variables in the regression, the results of the third-stage SFA regression can be obtained using Frontier 4.1 software. γ indicates the proportion of the variance of input slack values due to inefficient internal management or input scale in the total variance of input slack values. The larger the effect of internal management or input scale inefficiency on green economy efficiency, the larger the value of this statistic, which indicates that the adjustment of SFA for input variables is reasonable and necessary. For the environmental variables on input, redundancy is regarded as the opportunity cost of production and consumption in each province, that is, when the regression coefficient is positive, it leads to an increase in environmental variables and wastes inputs or decreases outputs, which is not conducive to improving green economic efficiency. Conversely, when the regression coefficient is negative, the increase of environmental variables saves inputs or increases outputs, which is conducive to improving green economic efficiency. The regression results are shown in Table 5.

**Table 5:** Regression results.

**Variables**	**Labor input slack variable**			**Capital input slack variables**			**Energy input slack variables**		
	**Labor force**	**Standard**	***t*-value**	**Energy**	**Standard**	***t*-value**	**Capital**	**Standard**	***t*-value**
	**factor**	**deviation**		**factor**	**deviation**		**Capital factor**	**deviation**	
INS	−0.111	0.120	−0.927	0.026	1.000	0.026	−0.053	0.051	−1.039
STR	0.000	0.001	−0.501	0.000	1.000	0.000	−0.001	0.000	−2.689
CITY	−0.023	0.100	−0.229	0.024	1.000	0.024	−0.004	0.043	−0.083
TEC	0.013	0.008	1.629	0.000	1.000	0.000	0.006	0.004	1.577
C	−0.168	0.104	−1.616	−0.034	1.000	−0.034	−0.067	0.049	−1.386
degama2	0.166	0.043	3.851	0.002	1.000	0.002	0.065	0.017	3.921
gama	0.905	0.026	35.394	0.290	1.000	0.290	0.950	0.014	70.119
Log function value	121.532			574.017			342.370		
LR test	404.226			242.068			388.508		

According to Table 5, the γ of energy input slack variables and labor input slack variables is 0.950 and 0.905, respectively, and both are significant at the 1% level, which indicates that management inefficiency is a major factor in each decision unit and needs to be adjusted as necessary. The coefficient of the energy structure of the energy input slack variable is significantly negative, which indicates that increasing the amount of coal consumption helps to reduce the redundancy of energy input. The coefficients of R&D input on labor input and energy input slack variables are significantly positive, indicating that reducing R&D input helps to reduce the redundancy of labor input and energy input. In summary, environmental factors have a significant effect on green economic efficiency.

There are differences in the effects of each environmental variable on different provinces, which may lead to better efficiency performance for some provinces facing a better external environment and worse efficiency performance for some provinces facing a worse external environment. Therefore, it is necessary to adjust the original input variables according to the regression results of the second stage, so that all provinces face the same external environment and thus obtain true energy efficiency.

#### Phase IV measurement results

**Figure 5:**
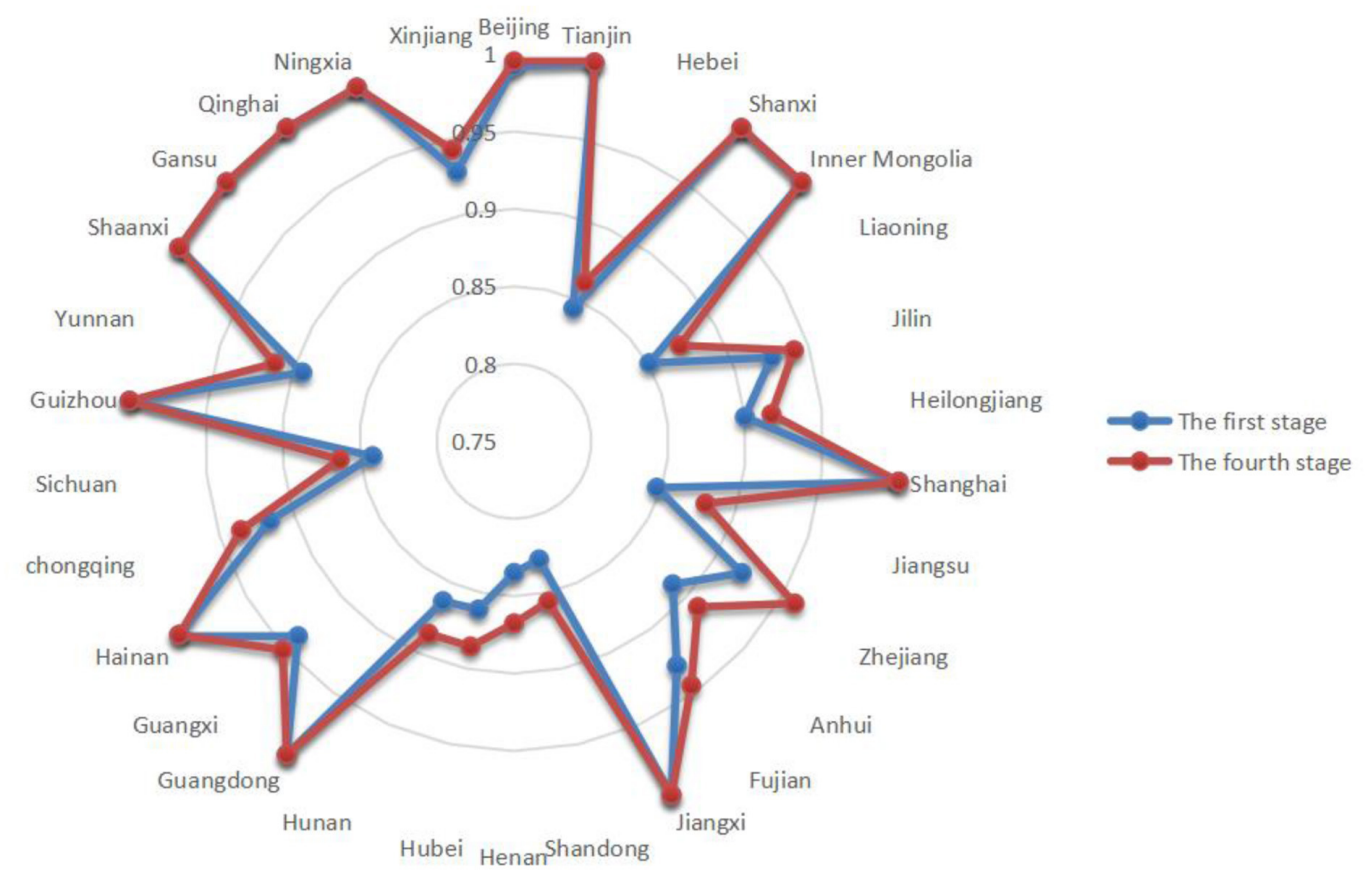
Comparison of the efficiency values of the first and fourth stages in 2011.

**Figure 6:**
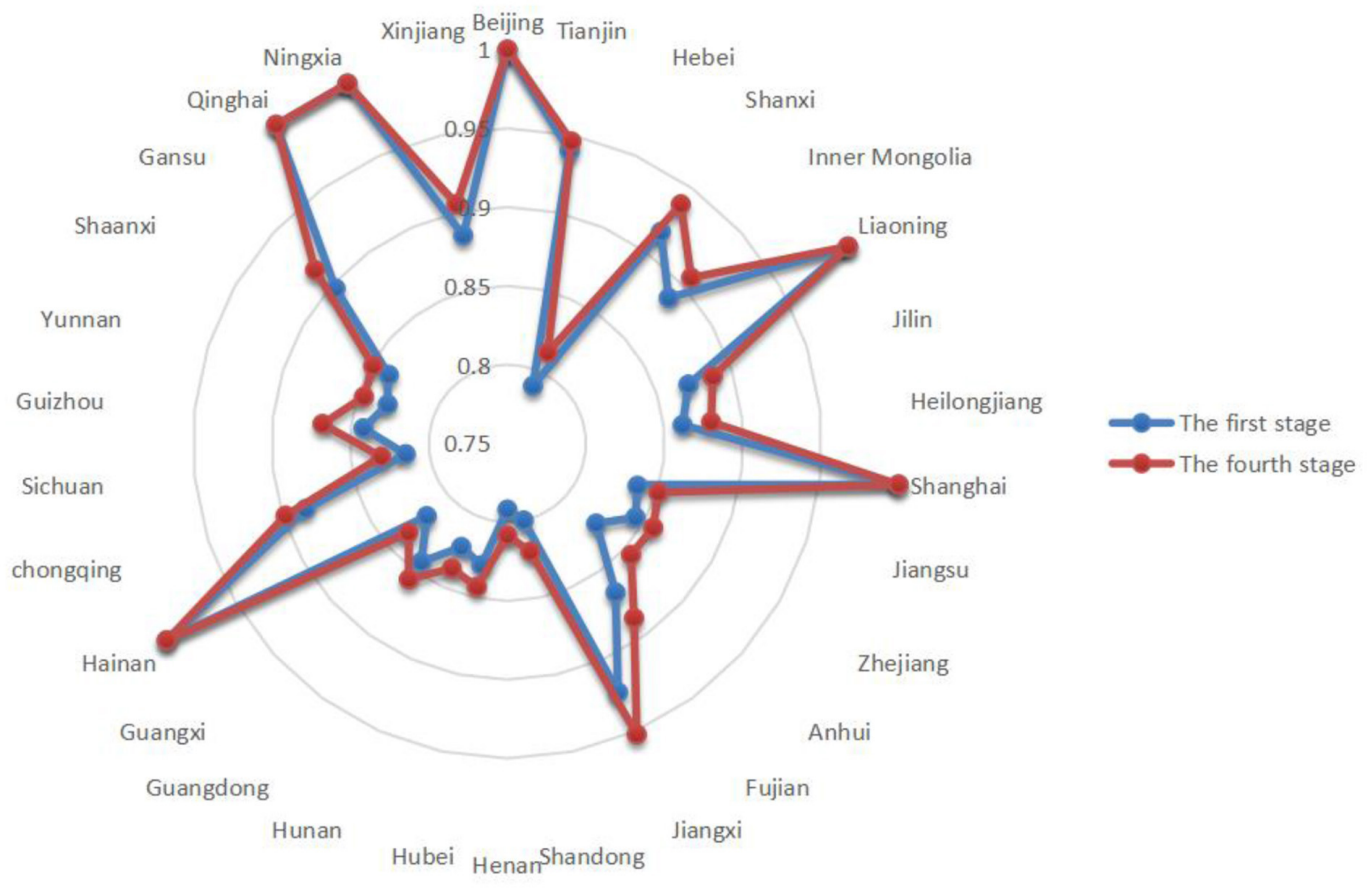
Comparison of efficiency values of the first and fourth stages in 2019.

The SBM-DEA model was again applied to measure the efficiency based on the adjusted amount of inputs and the initial amount of outputs in each province to obtain efficiency values that reflect the true internal management and input scale levels. The results are shown in Table 6, Figures 5, 6.

**Table 6:** Fourth stage.

**Province**	**2011**	**2012**	**2013**	**2014**	**2015**	**2016**	**2017**	**2018**	**2019**	**Aver**
Beijing	0.995	0.993	1.000	1.000	1.000	0.990	1.000	1.000	1.000	0.987
Tianjin	1.000	1.000	1.000	1.000	1.000	0.991	1.000	0.943	0.946	0.832
Hebei	0.862	0.850	0.844	0.836	0.824	0.821	0.820	0.813	0.813	0.915
Shanxi	1.000	0.912	0.878	0.870	0.859	0.857	0.963	0.956	0.938	0.930
Inner Mongolia	1.000	1.000	0.954	0.897	0.942	0.883	0.879	0.909	0.907	0.930
Liaoning	0.874	0.863	0.868	0.873	0.896	1.000	1.000	1.000	1.000	0.932
Jilin	0.940	0.920	0.954	0.952	1.000	0.935	0.931	0.871	0.887	0.899
Heilongjiang	0.917	0.900	0.895	0.908	0.922	0.896	0.892	0.879	0.880	0.999
Shanghai	1.000	1.000	1.000	0.998	1.000	0.998	0.994	1.000	1.000	0.858
Jiangsu	0.880	0.870	0.864	0.857	0.855	0.845	0.846	0.850	0.851	0.887
Zhejiang	0.959	0.917	0.902	0.894	0.880	0.857	0.858	0.861	0.857	0.870
Anhui	0.910	0.893	0.887	0.877	0.869	0.844	0.834	0.859	0.856	0.902
Fujian	0.945	0.923	0.921	0.901	0.905	0.891	0.864	0.879	0.887	0.978
Jiangxi	1.000	1.000	1.000	0.981	0.977	1.000	0.940	0.953	0.952	0.832
Shandong	0.856	0.849	0.851	0.842	0.826	0.815	0.816	0.809	0.820	0.833
Henan	0.868	0.857	0.852	0.844	0.837	0.809	0.807	0.809	0.808	0.858
Hubei	0.885	0.873	0.870	0.862	0.866	0.838	0.839	0.844	0.843	0.863
Hunan	0.886	0.877	0.878	0.868	0.885	0.851	0.849	0.838	0.837	0.911
Guangdong	1.000	1.000	1.000	0.883	0.874	0.866	0.862	0.860	0.857	0.887
Guangxi	0.951	0.930	0.931	0.917	0.894	0.850	0.840	0.836	0.835	1.000
Hainan	1.000	1.000	1.000	1.000	1.000	0.998	0.998	1.000	1.000	0.935
Chongqing	0.936	0.925	1.000	0.954	1.000	0.903	0.903	0.896	0.898	0.845
Sichuan	0.863	0.857	0.858	0.852	0.852	0.831	0.831	0.832	0.831	0.909
Guizhou	1.000	0.910	0.936	0.921	0.929	0.874	0.873	0.868	0.868	0.875
Yunnan	0.913	0.895	0.905	0.885	0.872	0.859	0.847	0.850	0.846	0.926
Shaanxi	1.000	1.000	1.000	1.000	0.918	0.859	0.855	0.855	0.848	0.911
Gansu	1.000	0.915	0.905	0.892	0.890	0.861	0.907	0.915	0.914	1.000
Qinghai	1.000	1.000	1.000	1.000	1.000	1.000	1.000	1.000	1.000	1.000
Ningxia	1.000	1.000	1.000	1.000	1.000	0.998	1.000	1.000	1.000	0.898
Xinjiang	0.942	0.919	0.902	0.889	0.875	0.878	0.869	0.905	0.905	0.998

The inter-provincial analysis shows that the green economic efficiency of 30 provinces varies to different degrees after excluding the influence of the external environment and random disturbances. This indicates that the external environment and random disturbances have a certain influence on the efficiency level, and the efficiency values measured by each province in the first stage are not very accurate. In general, the green economic efficiency gap is relatively obvious, with Guangxi, Gansu, and Qinghai having the highest average green economic efficiency, indicating that they have a reasonable layout in terms of pollution control and resource utilization efficiency. Shandong has the lowest average green economic efficiency, indicating that the province has a large gap in its green economic efficiency, and should focus on its resource utilization efficiency, environmental protection, and capacity planning.

Figures 5, 6 provide a comparison of green economic efficiency of 30 provinces in the first and fourth stages in 2011 and 2019, respectively, and it can be seen that after the adjustment of input indicators, the green economic efficiency of each province generally increases. The difference in green economic efficiency decreases from the first stage to the fourth stage, which indicates that the external environmental factors lead to a bias of efficiency estimation, and the influence of the environment on green economic efficiency can be removed through the adjustment of input variables.

### Results of panel model estimation of factors influencing green economic efficiency

The estimated results of the panel models are presented in Table 7, and the results of the individual fixed panel regressions with the gradual addition of control variables are presented in Models 1 through 5. The *p*-values for public health parameter estimates from Models 1 to 5 were all significantly negative at the 1% level. That is, the regression coefficient of public safety on green economic efficiency is always significantly negative. It shows that the occurrence of public security incidents will hinder green economic efficiency. The possible reason for this is that once a public safety event occurs, it can greatly affect everything from micro-individuals to macro-policies. A public safety event can bring about a series of chain reactions. Green industry development stagnates, green investment drops sharply, and human capital level decreases, which in turn greatly hinders green development and green economy efficiency decreases.

**Table 7:** Panel model regression results.

**Variables**	**Model 1**	**Model 2**	**Model 3**	**Model 4**	**Model 5**
PHS	−0.016***	−0.013***	−0.013***	−0.013***	−0.013***
	−4.080	−3.530	−3.550	−3.370	−3.510
GOV		−2.833***	−2.792***	−2.679***	−2.749***
		−5.600	−5.660	−5.670	−5.920
CON			−0.005	−0.001	−0.001
			−1.310	−0.180	−0.230
OPE				0.361***	0.328***
				3.370	3.070
PRI					0.059**
					2.150
C	1.012***	1.164***	1.196***	1.100***	0.837***
	266.770	41.200	27.290	23.710	6.420
Individual fixation	YES	YES	YES	YES	YES
Robustness test	YES	YES	YES	YES	YES
R2	0.748	0.782	0.783	0.789	0.793
*F*-value	246.020	182.360	179.320	267.160	193.010
NUMBER	270	270	270	270	270

^**^, ^***^ indicate significant at the 10, 5, and 1% levels, respectively.

The coefficient of the government's science and technology funding intensity is significantly negative, indicating that the increase in the government's science and technology funding intensity will hinder the efficiency of the green economy. The coefficient of foreign trade intensity is significantly positive, indicating that the increase of foreign trade intensity will promote green economy efficiency. The coefficient of energy price is significantly positive, indicating that the increase of energy price will promote green economic efficiency.

### Testing the moderating effect of environmental regulation

As shown in Table 8, the regression coefficient of the interaction term GUI^*^PHS on green economic efficiency is significantly positive at the 1% level. Specifically, a clean and hygienic environment not only prevents the occurrence of serious public health events but also prevents the spread of viruses and bacteria after such events. Environmental protection and public health have public goods characteristics (37, 38). In a market economy, it is difficult to solve the problem of negative pollution externalities by relying on the market alone, and the “invisible hand” of the government is needed to intervene in the market economy (47). When a public event has serious consequences, the government will adopt a corresponding mandatory environmental regulation policy. In the context of environmental regulation, companies will shift more funds to green research and development. Individuals will become more environmentally conscious and increase their green consumption. Investors will have a stronger preference for green projects and green business sectors and increase green investment. Thus, the efficiency of the green economy will be enhanced.

**Table 8:** Conditioning inspection results.

**Variables**	**Model 1**	**Model 2**
PHS	−0.009**	−0.024***
	−2.510	−5.450
GUI	0.008**	−0.001
	2.400	−0.160
PHS*GUI		0.007***
		3.720
C	0.753***	0.777***
	5.380	5.590
Control variables	YES	YES
Individual fixation	YES	YES
Robustness test	YES	YES
R^2^	0.796	0.805
*F*-value	94.670	101.400
NUMBER	270	270

^**^, ^***^indicate significant at the 10, 5, and 1% levels, respectively.

**Figure 7:**
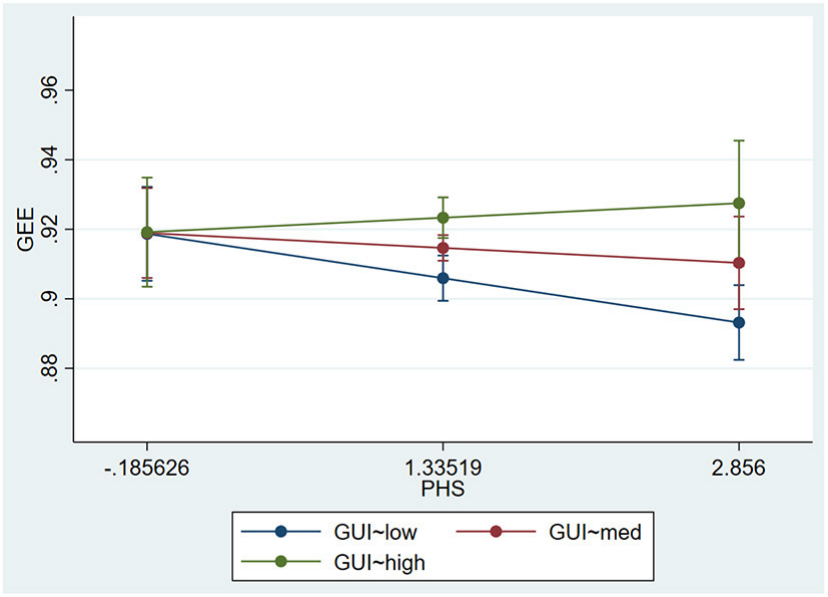
PHS → GEE.

Figure 7 depicts the impact of public health security on green economic efficiency under different levels of environmental regulation. As can be seen from Figure 7, the effect of public health events on green economic efficiency changes from negative to positive as the intensity of environmental regulation keeps increasing. That is, as the intensity of environmental regulation increases, the cost of pollution treatment faced by enterprises gradually increases, and the green preference of the whole market gradually increases. As a result, the speed of green transformation of enterprises accelerates, green R&D investment increases, green investment increases, and green economic efficiency keeps improving.

### Heterogeneity analysis

#### Geographic heterogeneity

**Figure 8:**
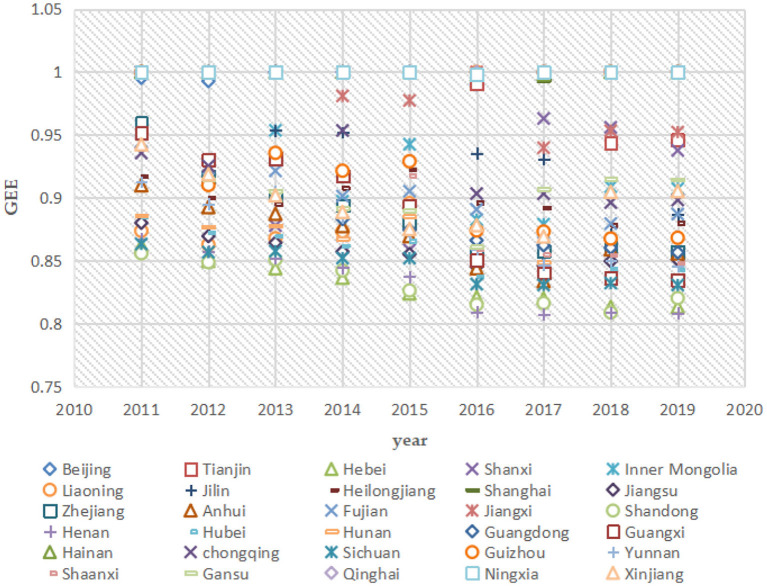
Green economic efficiency in 30 Chinese provinces, 2011 to 2019.

**Figure 9:**
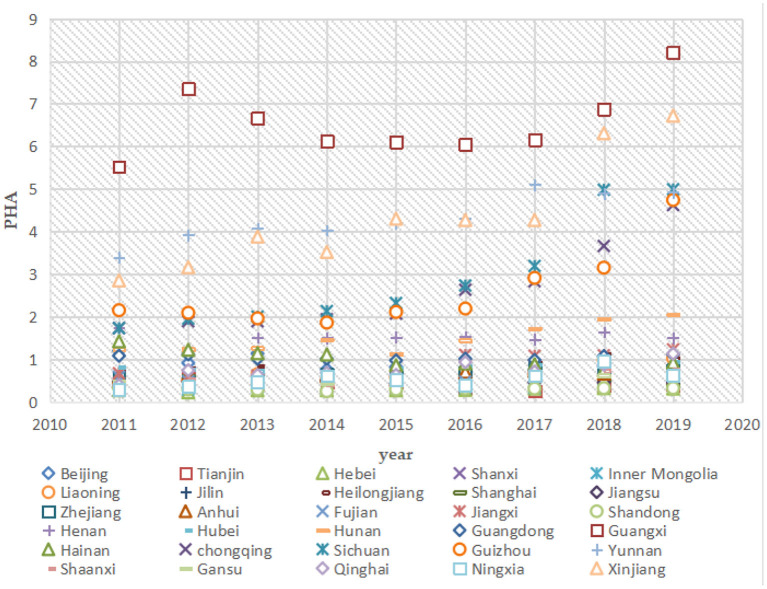
Public safety indicators for 30 Chinese provinces, 2011 to 2019.

China is a vast country with uneven spatial development, and there are large differences in green economic efficiency and public safety between provinces (e.g., Figures 8, 9). The impact of public safety on green economic efficiency may also be heterogeneous among different regions. To explore the regional heterogeneity of the impact of public safety on green economic efficiency, this paper divides the 30 provinces studied into eastern, central, and western regions according to the criteria of the three major economic zones of China's Seventh Five-Year Plan. The eastern region includes 11 provinces: Beijing, Tianjin, Hebei, Liaoning, Shanghai, Jiangsu, Zhejiang, Fujian, Shandong, Guangdong, and Hainan; the central region includes 8 provinces: Shanxi, Jilin, Heilongjiang, Anhui, Jiangxi, Henan, Hubei, and Hunan; and the western region includes 11 provinces: Guangxi, Sichuan, Chongqing, Guizhou, Yunnan, Shaanxi, Gansu, Qinghai, Ningxia, Xinjiang, and Inner Mongolia. The individual fixed-effects models were analyzed for the eastern, central, and western regions.

From Table 9, it is clear that the effect of public health events on green economic efficiency is most significant in the central part, followed by the eastern part, and not significant in the western part. In the central part, the coefficient of the effect of public health on green economic efficiency is−0.035 and is significant at the 1% level. In the east, the coefficient of the effect of public health on green economic efficiency is−0.017 and is significant at the 5% level. This indicates that the impact of public health events on green economic efficiency is geographically heterogeneous. The reason for the heterogeneity may be that the central region is at the geographic center of China, which is the intersection of people traveling from all provinces and is densely populated. When a public event occurs, the central region is most likely to be affected in terms of geographic distance, and thus green economic efficiency is most affected. This is followed by the eastern region. The eastern region is not in the middle, but is close to the coast. While being affected by security events in China, it is also affected by worldwide public security events, which in turn affect green economic efficiency. Lastly, the western region, which is deep inland, lags in development, has a slow movement of people, and thus has a limited impact on green economic efficiency.

**Table 9:** Heterogeneity of geographic location, carbon trading pilot, and traffic level.

**Variables**	**East**	**Central**	**West**	**Non-carbon pilot**	**Carbon pilot**	**Underdeveloped transportation**	**Moderately developed transportation**	**Well-developed transportation**
PHS	−0.017**	−0.035***	−0.002	−0.010 **	−0.001	−0.013	−0.005	−0.011**
	−2.140	−4.430	−0.370	−2.300	−0.010	−1.650	−0.790	−2.270
C	1.028***	0.917***	1.374***	0.897***	0.533	0.833***	0.951**	0.576*
	3.760	3.880	4.690	6.180	1.720	3.360	2.260	1.870
Control variables	YES	YES	YES	YES	YES	YES	YES	YES
Individual fixation	YES	YES	YES	YES	YES	YES	YES	YES
Robustness test	YES	YES	YES	YES	YES	YES	YES	YES
R2	0.880	0.884	0.827	0.814	0.905	0.832	0.774	0.900
Quantity	99	72	99	216	54	90	90	90

^*^, ^**^, ^***^indicate significant at the 10, 5, and 1% levels, respectively.

#### Carbon trading pilot heterogeneity

The original purpose of the carbon emissions trading market is to reduce pollution, and carbon trading has a significant impact on emissions, which has been proved in theory and practice (48–50). In the carbon trading pilot, the development of enterprises in the area is more likely to lead to them incorporating environmental protection into their corporate development strategies due to emission rights restrictions. Furthermore, the existence of the carbon trading pilot is to some extent propaganda for the green viewpoint, and the residents will reduce their consumption of high-pollution and high-emission products. Because of better environmental protection and sanitation, when a public health event occurs it has a less negative impact on the area and has less of a hindering effect on green economic efficiency. To explore the carbon pilot heterogeneity of the impact of public safety on green economic efficiency, this paper divides the provinces studied into carbon trading pilot areas, Beijing, Shanghai, Tianjin, Chongqing, Hubei, and Guangdong, according to the Notice on Carrying out the Pilot Work of Carbon Emission Trading issued by the National Development and Reform Commission of China in October 2011. Non-carbon trading pilot areas are Hebei Province, Shandong Province, Liaoning Province, Heilongjiang Province, Gansu Province, Jilin Province, Qinghai Province, Henan Province, Jiangsu Province, Hunan Province, Zhejiang Province, Jiangxi Province, Yunnan Province, Fujian Province, Hainan Province, Shanxi Province, Sichuan Province, Shaanxi Province, Guizhou Province, and Anhui Province. Individual fixed-panel regressions were conducted for carbon trading pilot and non-carbon trading pilot regions, as shown in Table 9. The effect of public safety on green economic efficiency is significantly negative in non-carbon trading pilot regions and insignificant in carbon trading pilot regions. That is, the occurrence of public events has a significant hindering effect on green economic efficiency in the non-carbon trading pilot. In the carbon trading pilot, the effect of public health event occurrence on green economic efficiency is not significant.

#### Traffic level heterogeneity

When a public safety incident occurs, the more convenient the traffic is, the more extensive and faster the public safety incident will be. In contrast, places with traffic congestion also control the extent and speed of public safety events to some extent because of the restricted time and distance for the movement of people and goods. Therefore, the impact of public health on green economy efficiency may be different in regions with different traffic levels. In this paper, individual fixed-panel regressions were conducted for each of the 30 Chinese provinces and divided into three groups: less-developed transportation, moderately developed transportation, and developed transportation. The regression results are shown in Table 9. It can be seen that the effect of public safety on green economic efficiency is not significant in both underdeveloped and moderately developed regions. The coefficient of the effect of public safety on green economic efficiency is−0.011 and significant at the 5% level in areas with developed transportation. That is, the more developed the transportation is, the stronger the hindering effect of public safety events on green economic efficiency.

## Robustness testing and endogeneity treatment

This paper uses several methods to test the robustness of the regression model. One is to perform a regression model turnover; the second is to replace the core explanatory variables; the third is to use the differential GMM method to address the endogeneity problem.

### Replacement regression model

To further verify the robustness of the conclusions of this paper, the pool model and random effects model are added to this paper, and the regression results are compared with individual fixed panel methods to test the robustness of our conclusions. The regression results of the models are shown for Model 1 and Model 2 in Table 10. It can be seen that the effect of public health on green economic efficiency is significantly negative, which supports the previous conclusion.

**Table 10:** Robustness tests.

	**Replacement**		**Substitution of core**		
	**regression model**		**explanatory variables**		
**Variables**	**Model 1**	**Model 2**	**Model 3**	**Model 4**	**Model 5**
PHS	−0.009^***^	−0.012^***^	−0.076^*^	−0.012^***^	−0.014^***^
	−4.700	−3.490	−1.740	−3.070	−3.910
C	0.890^***^	0.802^***^	3.583	0.847^***^	0.860^***^
	3.980	5.770	0.970	6.370	6.400
Control variables	YES	YES	YES	YES	YES
Individual fixation	NO	NO	YES	YES	YES
Robustness test	YES	YES	YES	YES	YES
R2	0.268	0.205	0.378	0.789	0.791
Quantity	270	270	270		

* and

*** indicate significant at the 10, 5, and 1% levels, respectively.

### Replacement of core variables

For the treatment of the explanatory variables, first, the super-efficient SBM-DEA model was used to measure green economic efficiency, and the results are shown in M3 in Table 10; second, to exclude the interference of outliers, regressions were conducted based on data from the 5 to 95% quartiles of green economic efficiency and public safety indicators, and the results are shown for M4 and M5 in Table 10. The above results indicate that the effect of public health on green economic efficiency is significantly negative, supporting the previous conclusion.

### Endogeneity test

In order to solve the endogeneity problem caused by bidirectional causality, this paper uses the systematic GMM estimation method to test endogeneity. The validity of the system GMM model setting depends on two preconditions: first, the disturbance term has significant first-order sequence autocorrelation; second, there is no second-order sequence autocorrelation. This paper reports the second-order serial autocorrelation test [AR(2) test] for all GMM regressions, and performs Hansen or Sargan tests. The null hypothesis of the serial autocorrelation test is that there is no serial autocorrelation, and the null hypothesis of the Hansen test and the Sargan test is that the instrumental variables satisfy exogeneity. To ensure model validity, the AR(2) test, Hansen test, or Sargan test with a P value greater than 0.05 is required. The specific regression results of the endogeneity test are shown in Table 11. The results of the autocorrelation test for all models show that AR(1) and AR(2) are always insignificant, and the first-order and second-order disturbance terms are not autocorrelated, indicating that the random terms of all models are not autocorrelated. The instrumental variables of each model pass the Sargan test, indicating that the selection of instrumental variables is reasonable. In addition, the estimated results of the main explanatory variables of the models are consistent and significant with the direction of the estimated coefficients of the benchmark model, further verifying that public health can have a significant impact on green economic efficiency.

**Table 11:** Endogeneity test.

**Variables**	**Model 1**	**Model 2**	**Model 3**	**Model 4**	**Model 5**
L.GEE	0.657^***^	0.605^***^	0.581^***^	0.481^***^	0.522^***^
	174.020	119.390	31.990	14.470	10.650
PHS	−0.003^***^	−0.004^**^	−0.002^***^	−0.007^**^	−0.006^***^
	−36.060	−11.110	−2.060	−3.250	−2.980
GOV		−0.147^***^	−0.444^***^	−0.600^***^	−0.637^***^
		−2.070	−3.520	−2.020	−2.880
CON			0.004^**^	0.004^***^	0.003^***^
			3.300	3.600	3.020
OPE				0.204^***^	0.158
				12.170	4.640
PRI					−0.004^***^
					−0.850
C	0.315^***^	0.367^***^	0.361^***^	0.448^***^	0.441^***^
	60.550	67.170	18.060	12.340	10.010
P-AR(1)	0.009	0.009	0.010	0.010	0.010
P-AR(2)	0.176	0.181	0.187	0.190	0.195
Sargan test	0.420	1.000	1.000	1.000	1.000
NUMBER	270	270	270	270	270

^**^, and ^***^indicate significant at the 10, 5, and 1% levels, respectively.

## Conclusions and insights

### Conclusions and innovation points

Public health has been a topic of great concern, especially in recent years. This article demonstrates whether there is an impact on public health green economy efficiency and the role of environmental regulation in achieving sustainable economic development. In theory, the occurrence of public security incidents will seriously hinder green economic efficiency. This paper empirically tested the path and heterogeneity of the impact of public health events on the efficiency of the green economy by constructing a panel model and an adjustment model.

The main conclusions of this paper are as follows: First, public health events have a significant hindering effect on the efficiency of the green economy. The occurrence of public health events will hinder the development of tourism, reduce the level of human capital, and hinder green investment, which will significantly affect the efficiency of the green economy. Second, environmental regulation plays a significant role in regulating the impact of public health events on the efficiency of the green economy. When a public security incident occurs, the government strengthens the intensity of environmental regulation to reduce the possibility of the recurrence of public health crises. With the increase in the intensity of environmental regulation, under the dual pressure of public crisis events and government mandatory policies, enterprises will choose green transformation, reduce pollutant emissions, and save resources for the sustainable development of enterprises, thus promoting green economic efficiency (34). Third, with the increase in the intensity of environmental regulation, the impact of public health events on the efficiency of the green economy has changed from hindering to promoting. That is, increased intensity of environmental regulation will speed up the green transformation of enterprises, encourage investment in green research and development, and continuously improve the efficiency of the green economy. Fourth, the impact of public health events on green economic efficiency is heterogeneous in terms of geographic location, carbon pilot, and transportation level.

There are three innovative points in this paper: First, it constructs green economic efficiency indicators by building a four-stage SBM-DEA model. Second, it explores the impact of public health events on green economic efficiency from the perspective of environmental regulation. Third, the impact of public health events on green economic efficiency is explored in terms of geographic location, carbon pilot, and traffic level heterogeneity.

### Insights

In the post-pandemic period, balancing economic development and environmental protection is urgently required. This paper provided the following insights: First, when a public safety event occurs, the government should quickly enter into prevention and control to stabilize the public and build self-confidence to prevent a sharp decline in green investment. Second, it is necessary to increase investment in human capital and improve people's physical resilience, and prevent a significant decline in the level of human capital when a public safety event occurs from hindering the efficiency of the green economy. Third, the intensity of environmental regulations should be increased in a reasonable way to reduce the hindering effect of public health events on green economic efficiency. Fourth, local governments should develop appropriate policies based on local conditions, taking into account local transportation levels, whether they are carbon pilots or their geographical locations.

### Research limitations

There are also certain research limitations in this paper. First, in the empirical part, it does not verify the transmission mechanism by which public health events affect the efficiency of the green economy through industrial structure, green investment, and human capital level. In the follow-up research, an intermediary model could be constructed to verify the transmission mechanism of public health events affecting green economic efficiency through industrial structure, green investment, and human capital level. Second, this paper does not discuss the spillover effect of public health events on the efficiency of the green economy. Public health events may affect the efficiency of the green economy in adjacent regions. Subsequent research could explore the spillover effect of public health events on green economic efficiency, and verify whether there is a spillover effect through aspatial econometric model.

## Data availability statement

The original contributions presented in the study are included in the article/supplementary material, further inquiries can be directed to the corresponding authors.

## Author contributions

Writing—original draft preparation: JZ. Writing—review and editing: YY, ZF, and KZ. All authors contributed to the article and approved the submitted version.

## Conflict of interest

The authors declare that the research was conducted in the absence of any commercial or financial relationships that could be construed as a potential conflict of interest.

## Publisher's note

All claims expressed in this article are solely those of the authors and do not necessarily represent those of their affiliated organizations, or those of the publisher, the editors and the reviewers. Any product that may be evaluated in this article, or claim that may be made by its manufacturer, is not guaranteed or endorsed by the publisher.
